# Sarcoidosis Presenting in Breast Imaging Clinic With Unilateral Axillary Lymphadenopathy

**DOI:** 10.7759/cureus.13245

**Published:** 2021-02-09

**Authors:** Thao Doan, Nga T Nguyen, Jing He, Quan D Nguyen

**Affiliations:** 1 School of Medicine, University of Texas Medical Branch, Galveston, USA; 2 Radiology, University of Texas Medical Branch, Galveston, USA; 3 Pathology, University of Texas Medical Branch, Galveston, USA

**Keywords:** sarcoidosis, unilateral lymphadenopathy, breast cancer, granulomatous disease, adenopathy, systemic disease

## Abstract

Sarcoidosis is an idiopathic systemic granulomatous disease that presents with noncaseating granulomas most commonly affecting the lungs and mediastinal lymph nodes. Patients often have nonspecific symptoms, including cough of unknown cause, fever, shortness of breath, fatigue, or weight loss. The diagnosis for sarcoidosis is relatively challenging in the sole presence of swollen lymph nodes and the absence of the aforementioned symptoms. We present a case of unilateral axillary lymphadenopathy found on routine mammography; ultimately proven to be an atypical symptom of sarcoidosis. Our goal is to highlight radiologic features that help distinguish sarcoidosis from potential malignancy.

## Introduction

Palpable lymph nodes when present are more related to benign than malignant disease processes [1]. The differential diagnosis for lymphadenopathy is broad and includes malignancy, inflammation, granulomatous disease, infection, medication, and iatrogenic causes [1]. A thorough history and physical examination are key to accurate diagnosis in most cases. In a subset of patients, unexplained lymphadenopathy whose cause cannot be readily diagnosable is suspicious of metastatic cancer, especially in the absence of infection or recent trauma.

Diagnosis for sarcoidosis requires data from clinical features, radiologic findings, histologic evidence of noncaseating granulomas, and exclusion of other causes. However, many patients are asymptomatic and thus are incidentally diagnosed with the presence of hilar lymphadenopathy during chest radiography. Sarcoidosis may also present with peripheral lymphadenopathy in approximately 2% to 25% of patients, which is also a common initial finding of several malignant diseases, such as breast carcinoma and lymphoma [2]. Because this is a systemic disease process, lymphadenopathy found in sarcoidosis cases is most commonly generalized and/ or bilateral. As a result, the presence of an isolated, unexplained swollen lymph node as presented in this case can make diagnosing sarcoidosis a challenging task, especially in the absence of other common systemic symptoms.

## Case presentation

**Figure 1:**
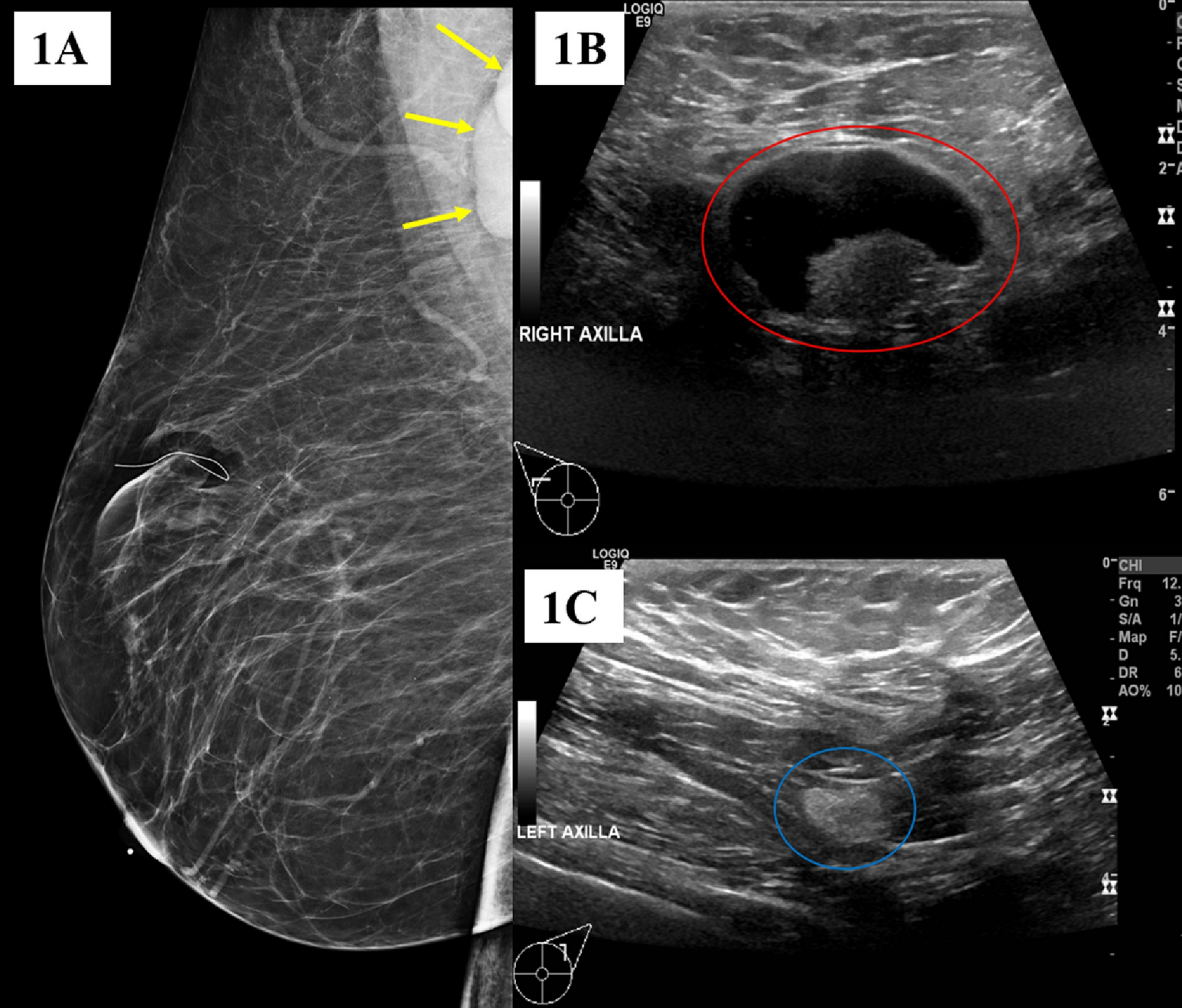
Screening Mammography of the Right Breast in Mediolateral Oblique View and Targeted Ultrasound of the Bilateral Axillae (A) The mammography image of the right breast shows axillary lymphadenopathy (yellow arrows). (B) The ultrasound image of the right axilla illustrates axillary lymphadenopathy measuring 35 mm with cortical thickening of 11 mm (red circle). (C) The ultrasound image of the left axilla shows a morphologically normal lymph node measuring 13 mm with a cortical thickness of less than 3 mm (blue circle).

A 66-year-old woman with a past medical history of pulmonary sarcoidosis presented for her routine screening mammography. Her family history was significant for a daughter having papillary carcinoma with bone metastasis. The patient self-reported as a past smoker. Her routine mammography showed scattered areas of fibro-glandular density in bilateral breasts and right axillary lymphadenopathy (Figure 1A). There was no evidence of suspicious masses, calcifications, or other abnormal findings in the right and left breasts. Targeted ultrasound of the axillary, supraclavicular, infraclavicular, and internal mammary regions showed an enlarged axillary lymph node in the right axilla, measuring 35 mm in diameter with diffuse cortical thickening of 11 mm (Figure 1B). A normal lymph node was also found in the left axilla (Figure 1C).

**Figure 2:**
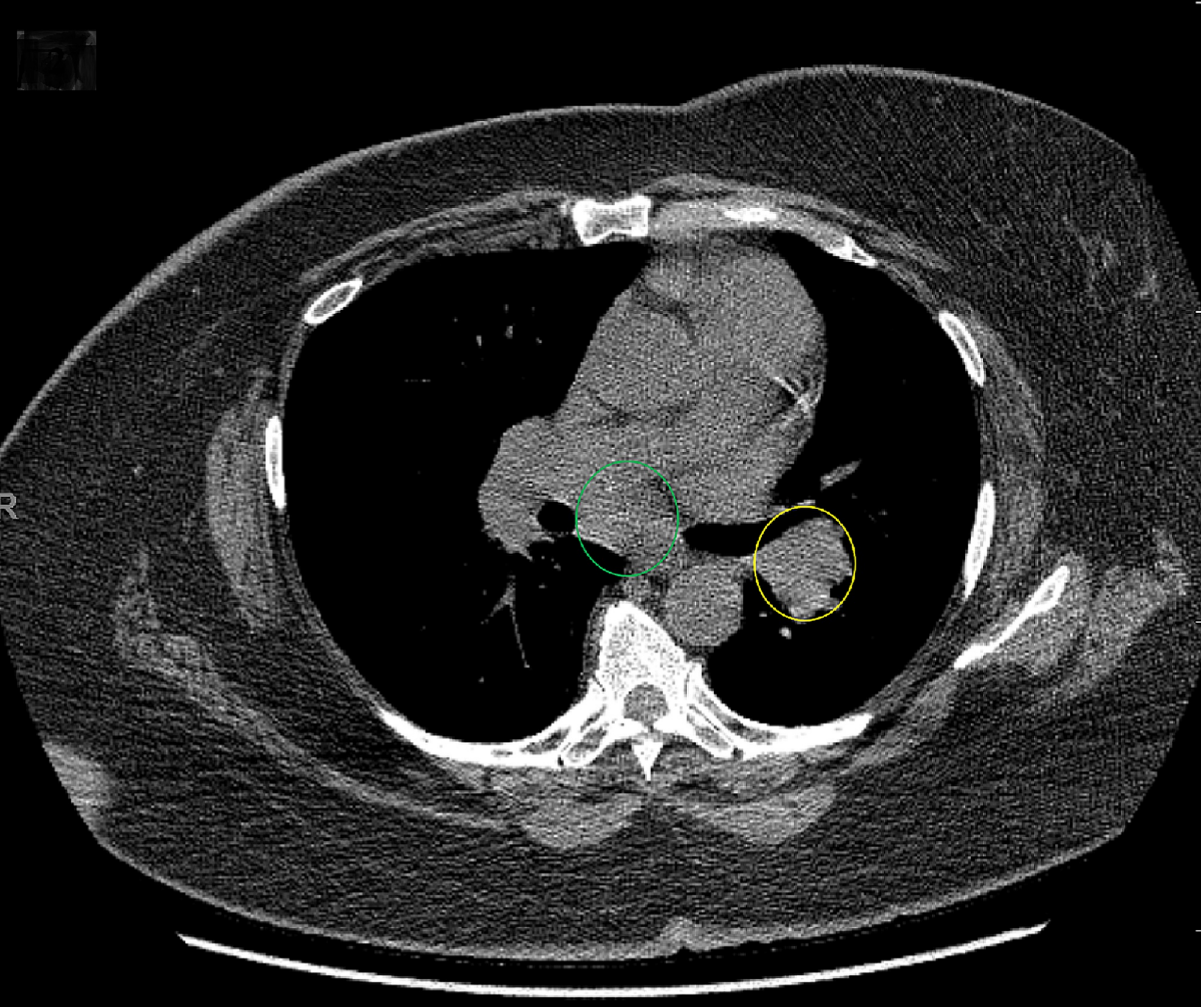
Axial Computed Tomography Scan of the Chest in Soft Tissue Window Prominent hilar (yellow circle) and mediastinal (green circle) lymphadenopathy is seen.

**Figure 3:**
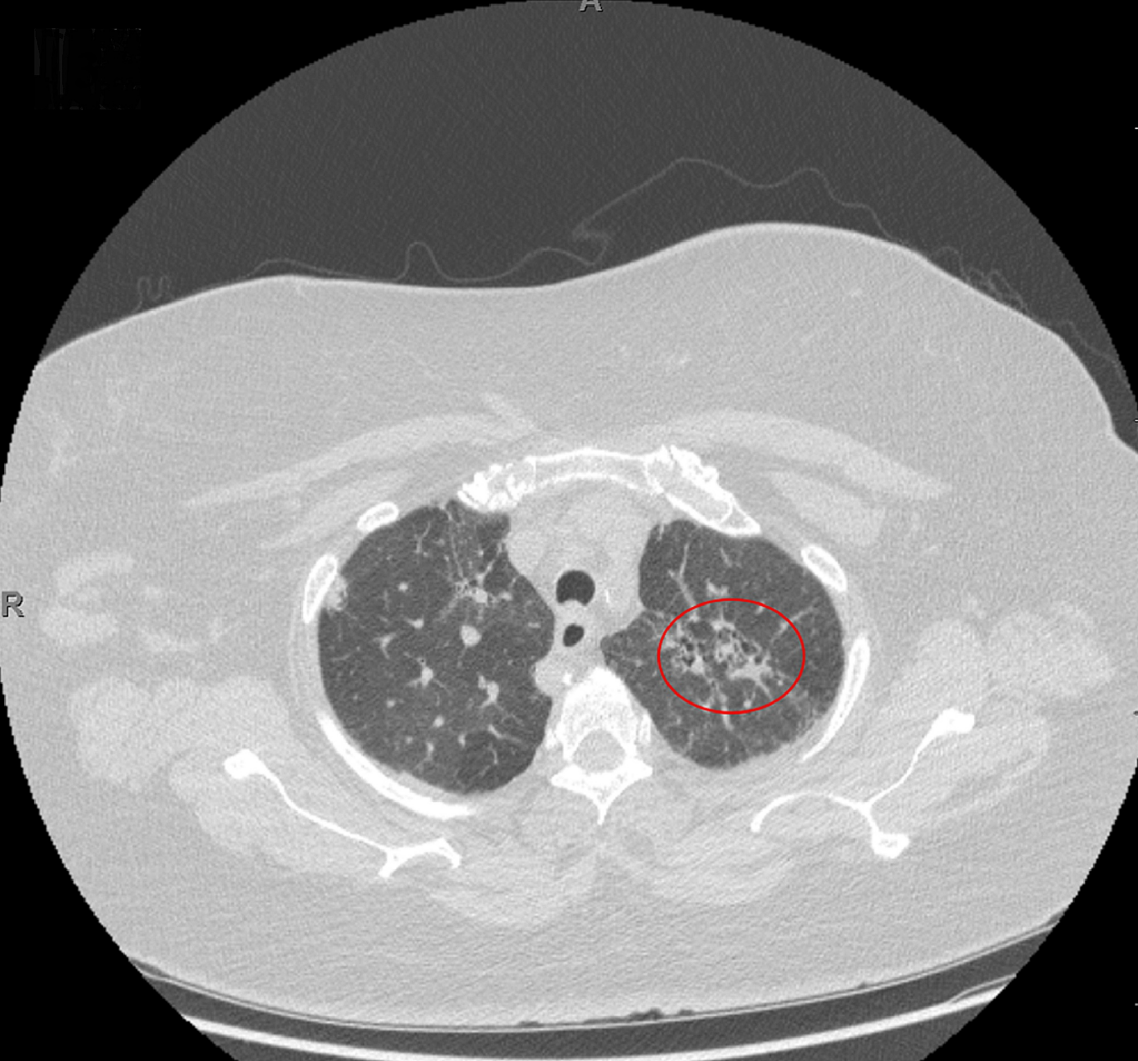
Axial Computed Tomography Scan of the Chest in Lung Window Interlobular septal thickening and traction bronchiectasis with foci of honeycombing are present in the upper lobe (red circle) along with scattered ground-glass opacities.

**Figure 4:**
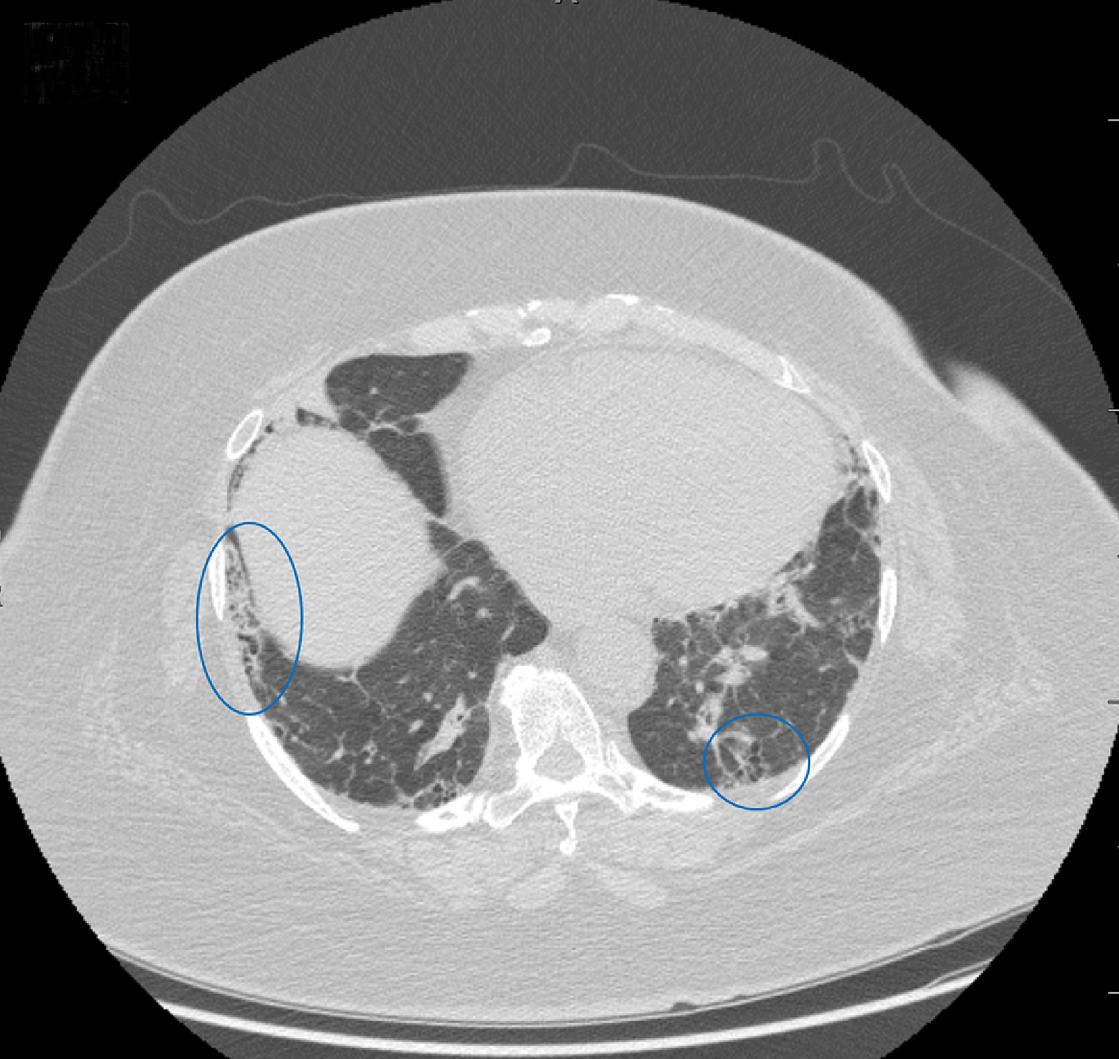
Axial Computed Tomography Scan of the Chest in Lung Window Bilateral irregular subpleural reticulation is present in the lower lobes (blue circles), along with scattered ground-glass opacities.

Due to the patient's past medical history of sarcoidosis and the suspicion of malignancy, a CT scan of the thorax was performed to further evaluate the accurate diagnosis. The CT scan of the chest showed hilar and mediastinal lymphadenopathy (Figure 2). Bilateral irregular subpleural reticulation with interlobular septal thickening and traction bronchiectasis with foci of honeycombing was seen predominantly in the upper and lower lobes, along with scattered ground-glass opacities (Figures 3, 4).

**Figure 5:**
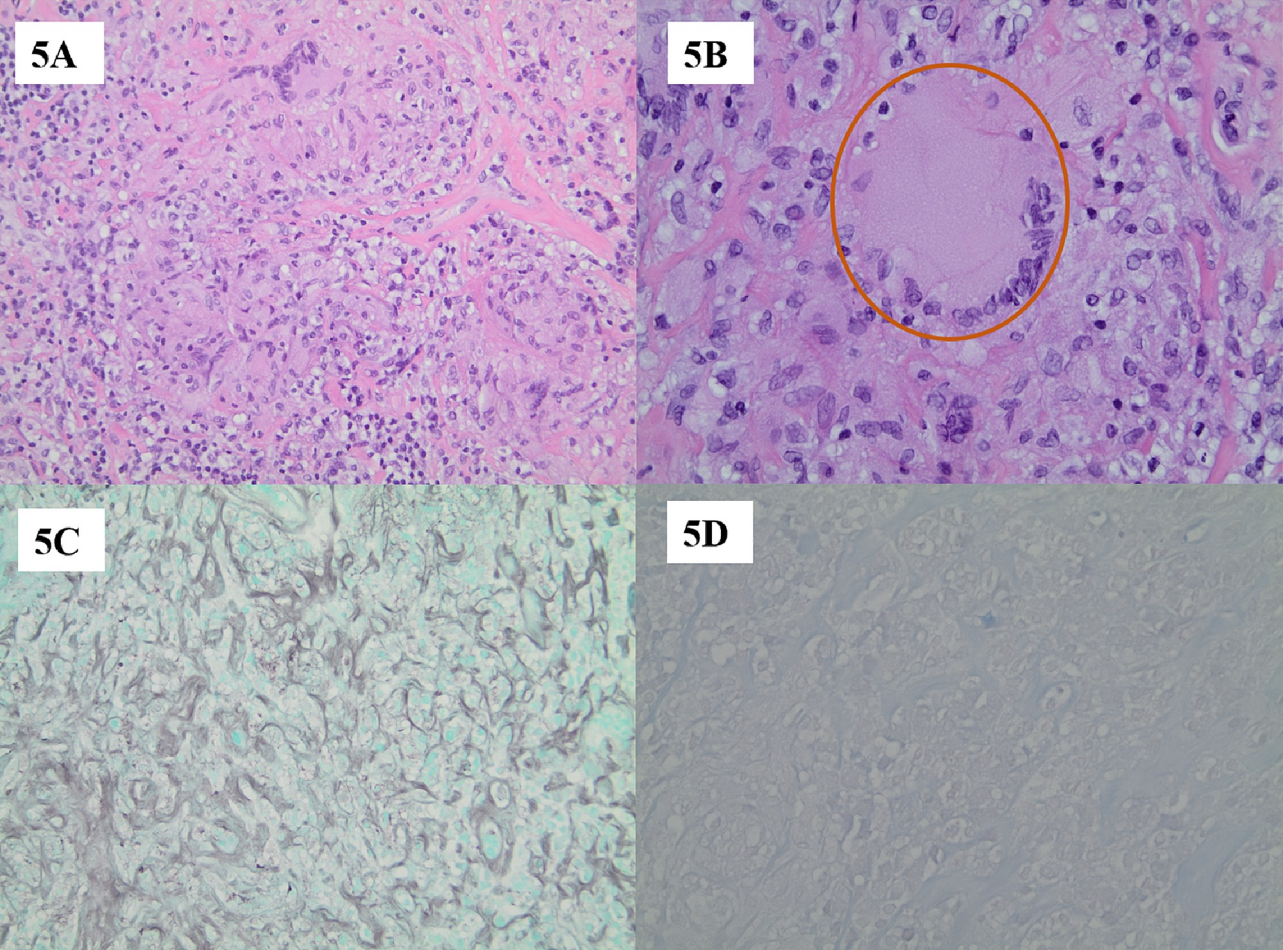
Histological Findings of Non-necrotizing Granulomatous Inflammation (A) Histological findings illustrate non-necrotizing granulomatous inflammation. Necrosis is absent. This is demonstrated at a magnification of 200x. (B) Epithelioid cells with scattered Langerhans giant cells (brown circle) and lymphocytes are present. This is demonstrated at a magnification of 400x. (C) Grocott methenamine silver stain shows negative staining for fungal organisms. This is demonstrated at a magnification of 200x. (D) Acid-fast bacilli stain shows negative staining for acid-fast organisms. This is demonstrated at a magnification of 400x.

A tissue biopsy of the right axillary lymph node was performed for a definitive diagnosis. The histological findings showed non-necrotizing granulomas composed of epithelioid cells with scattered Langerhans giant cells and lymphocytes (Figures 5A, 5B). Staining for acid-fast bacillus and fungus was negative (Figures 5C, 5D).

The above findings were consistent with her history of pulmonary sarcoidosis and hence, she was recommended to follow-up with her pulmonologist.

## Discussion

The presence of generalized lymphadenopathy almost always indicates a significant systemic disease process, such as lymphoma, sarcoidosis, mononucleosis-type syndromes, tuberculosis, human immunodeficiency virus infection, and hepatitis. On the other hand, localized lymphadenopathy means only one area is involved and often indicates a local infection or malignancy [3]. Oftentimes, the anatomic location of localized adenopathy may be helpful in the diagnostic process. The most useful diagnostic hint is the presence of tumor, infection, or skin lesions in the region drained by the nodes. In this particular case, the patient presented with a unilateral swollen axillary lymph node that drains the walls of the thorax, the arm, the breast, and the upper abdominal wall. Nevertheless, she did not have any active symptoms of fevers or chills, breast masses, or skin lesions. Therefore, reactive adenopathy is less likely. Because the patient has a history of pulmonary sarcoidosis, extrapulmonary manifestation with peripheral lymphadenopathy was likely. However, she did not have any active symptoms of sarcoidosis on presentation. Hence, her differential diagnosis included multiple malignant and benign disease processes, such as granulomatous disease, breast cancer, lymphoma, autoimmune causes, or reactive infectious diseases. Many of these conditions present with similar constitutional symptoms, such as fever, fatigue, and weight loss. As a result, further imaging, fine needle aspiration, and tissue biopsy were necessary for a definitive diagnosis.

Sarcoidosis can be confused with many other diseases, especially in the absence of its typical presentations, such as cough, constitutional symptoms, uveitis, and erythema nodosum. On imaging, 90% of the cases show hilar, paratracheal, and mediastinal lymphadenopathy which is often bilateral and can progress to interstitial lung disease [4]. Peripheral lymph nodes can be found in up to 25% of the patients with the most common locations being cervical, supraclavicular, and inguinal regions [2]. Sarcoidosis axillary adenopathy, as in this case, is found in only 11.9% of the patients with peripheral lymph node involvement [2]. Cases of axillary sarcoidosis have been reported in the literature. However, unlike our case with single axillary adenopathy, lymphadenopathy in those cases involved other areas, such as supraclavicular and cervical regions [5,6].

Malignancy is an important differential diagnosis to rule out in the sole presence of adenopathy. Malignant causes of axillary lymphadenopathy include breast cancer, lymphoma, melanoma, or metastases from other sites. Metastatic breast cancer may present with an initial spread to bilateral anterior and central axillary lymph nodes [7]. Imaging of breast cancer often demonstrates irregular-shaped breast masses, increased nodal density, loss of fatty hilum, or micro-calcifications [8]. Additionally, ultrasound often shows enlarged lymph nodes with increased intralesional vascularity. Although cancer is an important diagnosis to be evaluated for, studies have found that the risk of malignancy with lymphadenopathy is very low [1]. Additionally, the incidence of breast cancer presenting with metastases to axillary lymph nodes in the absence of a breast tumor is less than 1% [8]. However, axillary lymphadenopathy found on a mammogram is associated with more malignant than benign cases [8]. As a result, the low risk of malignancy with isolated lymphadenopathy does not lessen the importance of identifying the accurate diagnosis when a patient presents with localized adenopathy of an unknown cause. In this case, because of the presence of unexplained unilateral lymphadenopathy on imaging and several other risk factors, including her smoking history, family history of metastatic cancer, and advanced age, further investigation with ultrasonography, CT scan, and ultrasound-guided tissue biopsy were warranted.

Diagnosis for sarcoidosis requires combined information from clinical features, radiologic findings, and histologic evidence. Sonographic findings of axillary sarcoidosis include hypoechoic, oval, or round lymph nodes with well-circumscribed margins and a homogenous echotexture [9]. The aforementioned features are non-specific and hence, may overlap with metastatic cancer and lymphoma [10]. The ultrasound of the swollen lymph node may also show disruption of the nodal architecture, which resembles a “zebra pattern” but this unique finding has not been consistently correlated with sarcoidosis in the current literature [10]. Due to the high likelihood of pulmonary involvement, biopsies of the lungs and mediastinal lymph nodes are commonly used to confirm the initial diagnosis of sarcoidosis [4]. Histopathological findings of sarcoidosis include non-necrotizing granulomatous inflammation with the presence of epithelioid and multinucleated giant cells. In addition to imaging and histopathological findings, bronchoalveolar lavage with an increased CD4:CD8 T-lymphocyte is also a useful diagnostic tool. Additionally, there are currently no reliable biomarkers for sarcoidosis in clinical practice. Although a large percentage of patients have elevated angiotensin-converting enzyme (ACE) levels, there is still variability among individuals. As a result, ACE levels do not correlate with the severity of disease and lack adequate specificity for diagnosis confirmation and sensitivity for screening [11].

## Conclusions

Sarcoidosis lymphadenopathy often presents bilaterally in multiple regions. As a result, the unilaterality of the lymph node involvement and the absence of active sarcoidosis symptoms in our case can make the accurate diagnosis a challenging task. The differential diagnosis for unilateral axillary adenopathy is broad, including both benign and malignant causes. Malignancies such as metastatic breast cancer and lymphoma must be excluded. Therefore, multiple imaging modalities and tissue biopsies are warranted to assist with the diagnostic process.
